# Polarization-Accelerated Seawater Splash Simulation for Rapid Evaluation of Protection Performance of an Epoxy Coating on Carbon Steel

**DOI:** 10.3390/ma17143623

**Published:** 2024-07-22

**Authors:** Yuqing Xu, Guangling Song, Dajiang Zheng, Changsheng Liu, Enhou Han

**Affiliations:** 1 School of Chemistry and Chemical Engineering, Guangzhou University, Guangzhou 510006, Chinaehhan@gzhu.edu.cn (E.H.); 2 Center for Marine Materials Corrosion and Protection, Xiamen University, Xiamen 361005, China; 3 Institute of Corrosion Science and Technology, Guangzhou 510070, China; 4 Department of Ocean Science and Engineering, Southern University of Science and Technology, Shenzhen 518055, China; 5 School of Materials Science and Engineering, Northeastern University, Shenyang 110819, China

**Keywords:** degradation acceleration, AC-DC-AC, ocean environment, epoxy coating, steels

## Abstract

The application of organic coatings is the most cost-effective and common method for metallic equipment toward corrosion, whose anti-corrosion property needs to be improved and evaluated in a short time. To rapidly and rationally assess the anti-corrosion property of organic coatings in the ocean splash zone, a new accelerated test was proposed. In the study, the corrosion protection property of the coating samples was measured by an improved AC-DC-AC test in a simulated seawater of 3.5 wt.% NaCl solution, a simulated ocean splash zone test and a new accelerated test combining the above two tests. The results showed that the corrosion rate of the coating samples was high in the improved AC-DC-AC test, which lost its anti-corrosion property after 24 cycles equal to 96 h. The main rapid failure reason was that the time of the water and corrosive media arriving at the carbon steel substrate under the alternating cathodic and anodic polarization with symmetrical positive and negative electric charges was shortened. The entire impedance of the coating samples was improved by about 1.6 times more than that in the initial early time in the simulated ocean splash zone test, which was caused by the damage effect from the salt spraying, drying, humidifying, salt immersion, high temperature and UVA irradiation being weaker than the enhancement effect from the post-curing process by the UVA irradiation. In the new accelerated test, the samples lost their corrosion resistance after 12 cycles equal to 288 h with the fastest failure rate. On account of the coupling process of the salt spraying, drying, humidifying, salt immersion, high temperature combined with the cathodic and anodic polarization and the UVA irradiation, the penetration and transmission rate of water and corrosive media in the coating were further accelerated, the corrosion rate on the carbon steel substrate was reinforced even larger and the destruction of the top polymer molecules was more serious. The new accelerated test showed the strongest damage-acceleration effect than that in the other two tests.

## 1. Introduction

The global economic loss from corrosion is huge for metallic structures in harsh ocean environments. To prevent the damage by prolonging the service life of numerous metal equipment, the application of organic coatings is the most cost-effective and widespread way [1,2,3,4,5]. It is crucial to evaluate the corrosion resistance of the organic coating systems under the actual exposure environment. However, the test process for a heavy-duty coating is normally very time consuming and costly. It is imperative to exploit accelerated tests to rapidly assess the degradation behavior and anti-corrosion property of such coating systems [6].

Currently, the majority of accelerated tests are used indoor to simulate the service environments, such as the single neutral salt-spray (ASTM B117 [7]) [8], the Prohesion test [9,10], the QUV (ASTM G154 [11]) test [12] and the Prohesion combined with QUV (ASTM D5894 [13]) test [14,15]. Although these aforementioned standard tests have the capability to replicate certain authentic environmental factors, like salt and light radiation, they are still relatively too simple to really simulate some complex service environments. Therefore, it is essential to establish a multi-factor coupling-accelerated test to research the failure behavior of coating systems in harsh ocean environments [16]. 

For the splash zone [17,18,19] of the worst ocean environment, the environmental factors should at least include salt, ultraviolet (UV) radiation [20,21], heating and wet–dry cycling. In the previous literature, it is common to simulate the environment by alternating wet and dry [22,23,24] or soaking [25,26], which failed to include the above major environmental factors. Hence, it is indispensable to design a new corrosion-accelerated test that includes the above environmental factors. Up to now, the cyclic corrosion test contains salt spray, humidity and dry cycles with different cycle durations, temperatures and humidity levels [27,28,29] which should be absorbed in the new corrosion-accelerated test. 

However, another problem is that the designed multi-factor coupling simulation test may not guarantee a fast failure rate for the coatings, apart from the lack of quantitative electrochemical corrosion information, although it may better simulate the actual environment. Therefore, it is still necessary to further find a more rapid acceleration method for the failure of the coatings.

Nowadays, Electrochemical Impedance Spectroscopy (EIS) is widely applied to monitor the mutations of protective performance of coatings in aggressive solutions, as a kind of sensitive, non-destructive, fast and in situ technique [30,31]. That would be a great technique to combine EIS with accelerated failure techniques for invigilating the mutations that occur in the coating/metallic substrate systems [32,33,34], which was first proposed by Hollaender and co-workers [35,36]. The method, called AC-DC-AC, includes three procedures in each cycle: firstly, an EIS measurement (AC) is carried out to monitor the original corrosion resistance of organic coating systems; then, a cathodic polarization (DC) is applied to accelerate the rate of hydrogen evolution and alkalization at the interface between the coating and metallic substrate; after, the coating system is relaxed until it attains a new state of equilibrium; then, another EIS measurement is conducted to assess the damage condition of the organic coating system in the back of the DC polarization. The method has also been successfully employed to efficiently assess the corrosion resistance of liquid coatings [37,38] powder paints [39], cataphoretic coatings [40], UV paints [41] and anodic thin films [42]. Nowadays, the technique has advanced to an accelerated cyclic electrochemical technique, named ACET corresponding to UNE-EN ISO 17463:2014 [43].

In order to be more reasonable in accelerating the organic coatings as in the natural environments, some modified AC-DC-AC techniques have been put forward [44,45,46,47,48]. Especially, in our group’s last research [48], the corrosion damage of the epoxy coating samples could be rationally accelerated by the modified AC-DC-AC technique to mimic that in the natural immersion environment. The technique was applied to one cycle in a 24 h period, whose DC step contained cathodic and anodic polarization with symmetrical positive and negative electric charges during the anodic and cathodic half-cycles, respectively. This will also be a good inspiration for solving the problem that the acceleration efficiency is not high enough.

In this article, three different corrosion-accelerated tests were implemented to assess the anti-corrosion property and study the corrosion behavior of epoxy coating systems. The first test was an improved AC-DC-AC corrosion-acceleration mean, the second simulated the ocean splash zone test and the third test was a combination of the first and second tests. It is expected that the combination test can more rapidly and rationally evaluate the anti-corrosion property of epoxy organic coatings in the ocean splash zone, and gain a deeper understanding of the interplay between the electrochemical polarization and the multi-factor environmental simulation towards the deterioration of a epoxy coating system.

## 2. Experimental 

### 2.1. Materials

**Figure 1:**
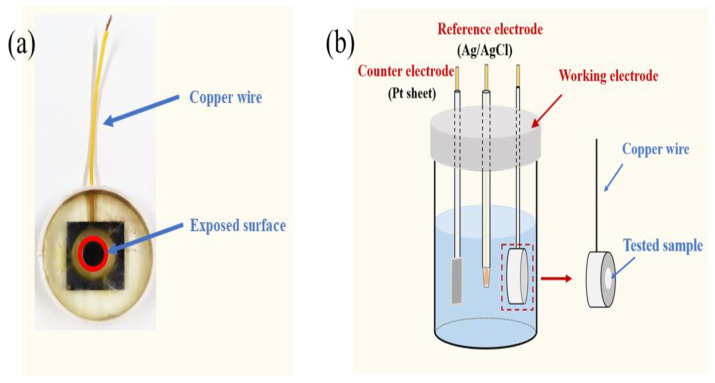
The tested sample figure (**a**) and schematic illustration of the three-electrode cell used for EIS test (**b**).

The epoxy coating, consisting of glycidyl ester epoxy resin and fatty amine hardener, was purchased from Shanghai Tengyi Industrial Co., LTD. (Shanghai, China). The substrate carbon steel panels were procured from Dongguan Shanghai special steel Co., LTD with the shape of 25 mm × 25 mm × 2 mm. Its chemical compositions were 0.44% C, 0.62% Mn, 0.015% P, 0.22% Si, 0.009% S, 0.01% Cr, 0.028% Al and balance Fe (weight fraction). Before the epoxy coating application, the hardener was added to the resin for full mixing with the weight ratio of 1:4 for hardener and resin. And then, the mixture was spun onto the carbon steel surface after polishing it with 800 mesh sandpaper. The glue machine named EZ4 was purchased from Jiangsu Leibo Scientific Instrument Co. LTD. (Wuxi, China). After that, the samples were placed in a dust-free environment at room temperature and cured for 2 h, and then were placed in an oven for 12 h of curing at 90 °C. The average thickness of the dry samples (see Figure 1a) was about 12 ± 0.5 µm, which was estimated with an elcometer (Fisher, Waltham, MA, USA, MPO).

### 2.2. EIS Test

A three-electrode cell (see Figure 1b) was used to perform the EIS tests, which contained the reference electrode of an Ag/AgCl (3.5 M KCl, R0303), the counter electrode of a platinum foil (15 mm × 15 mm × 0.2 mm) and the working electrode of the sample. The exposed area in 3.5 wt.% NaCl solution of the sample was about 1.0 cm^2^. The distance between the sample and the Ag/AgCl was 10 mm, the same as the Ag/AgCl and the platinum foil. All the EIS tests were placed in a metallic cage for avoiding external electromagnetic interference.

The EIS tests were conducted using a Gamry Interface potentiostat (1010E) coupled with a frequency response analyzer, while maintaining an open circuit potential (OCP). The frequency range employed in the EIS tests spanned from 10^5^ Hz to 10^−2^ Hz, and the sinusoidal voltage was 10 mV (rms). The corrosion protection performance of the coating system is directly correlated with its resistance. Numerous studies have employed the total impedance at the lowest frequency to gauge the extent of destruction in coating systems. When this total impedance falls below the threshold value of 1.0 × 10^7^ Ω·cm^2^, it signifies a loss in protective efficacy for the coating system [49,50,51]. All the impedance analysis was executed as the standard of ISO/TR 16208-2014 [52].

### 2.3. Three Accelerated Tests

The samples researched in this article were placed into three accelerated tests with cyclic mode. Details of the measurement condition and process of each test are summarized as follows:

(1) An improved AC-DC-AC test including alternant cathodic and anodic polarization with symmetrical positive and negative electric charges, called E_Polarization+Immersion_. This test contained four parts. (a) The samples were immersed in the 3.5 wt% NaCl solution with the first OCP step for time t_1_. (b) Then, an AC EIS test was put into effect for time t_2_. (c) Then a cathodic DC (polarization) with −0.05 mC under −4 V was applied by using the Chronocoulometry method, and then an anodic DC (polarization) with 0.05 mC under 4 V was applied equally. Among these, the −4 V used in the cathodic DC (polarization) was derived from the accelerated cyclic electrochemical technique, named ACET corresponding to UNE-EN ISO 17463:2022 [53]; as a comparison goal, 4 V was also applied. The time of this step was t_3_. (d) Then, the sample was measured with the second OCP step for time t_4_. The total time (t_1_ + t_2_ + t_3_ + t_4_) of one cycle in this test was 4 h.
(2) An alternating salt spray (containing wet, dry and humid), immersion and UV irradiation procedure was applied to simulate the ocean splash environment, called E_Salt+Immersion+UV_. This test contained three parts. (a) The samples were stuck in a salt spray container under a wet condition (5 ± 0.5 wt.% NaCl solution, 35 ± 2 °C, pH: 6.5~7.2, continuous spray) for 2 h. Then, the samples were transferred into a steady temperature and humidity chamber under dry conditions of 60 ± 2 °C and 25 ± 5% humidity for 4 h, and then under wet conditions of 50 ± 2 °C and >95% humidity for 2 h. (b) Then, the samples were immersed in the 3.5 wt% NaCl solution with an OCP step for 4 h. (c) Then, the samples were taken out and exposed to the ultraviolet irradiator under an irradiance of 0.89 W/m^2^/nm in the container for 12 h. The entire time of one cycle under this test was 24 h.
(3) Combination of the E_Polarization+Immersion_ test and the E_Salt+Immersion+UV_ test, called E_Salt+Polarization+Immersion+UV_. This new test contained three parts. (a) The step was the same as in the (a) of the (2) in the E_Salt+Immersion+UV_ test, so the time was 8 h. (b) Then, the step was the same as in the (1) of the E_Polarization+Immersion_ test, so the time was 4 h. (c) Then, the step was the same as in the (c) of the (2) in the E_Salt+Immersion+UV_ test, so the time was 12 h. The entire time of one cycle in this test was also 24 h.

To ensure the reproducibility of the results, at least three parallel samples were tested under the same conditions in the three accelerated tests.

### 2.4. Coating Characterization

The surface morphology of the epoxy coating samples was observed using a Leica DVM6 microscope (Wetzlar, Germany). The surface roughness of the samples was detected using a laser confocal scanning optical microscope (LCSOM, Keyence VK-X200, Osaka, Japan). A portable colorimeter (WR-10QC, Shenzhen, China) was utilized for calculating the color difference and reflection spectrum of the samples. The micro-FTIR spectra were measured in the wave number range from 4000 cm^−1^ to 600 cm^−1^ using a Nicolet iS50 FTIR spectrometer (Thermo Scientific Inc., Waltham, MA, USA), enabling analysis of the chemical structure of the coating samples. The adhesion between the epoxy coating and carbon steel substrate was evaluated utilizing a PosiTest pulled-off adhesion tester with a diameter of 10 mm and an applied stress rate of 0.25 MPa/s. Scanning electron microscopy (SEM) and energy dispersive spectroscopy (EDS) were employed on a Phenom XL instrument (Shanghai, China) to capture local images of corroded areas on the carbon steel substrate as well as measure Fe content and O content after adhesion testing at an operating voltage of 15 kV.

## 3. Results

### 3.1. EIS Results

**Figure 2:**
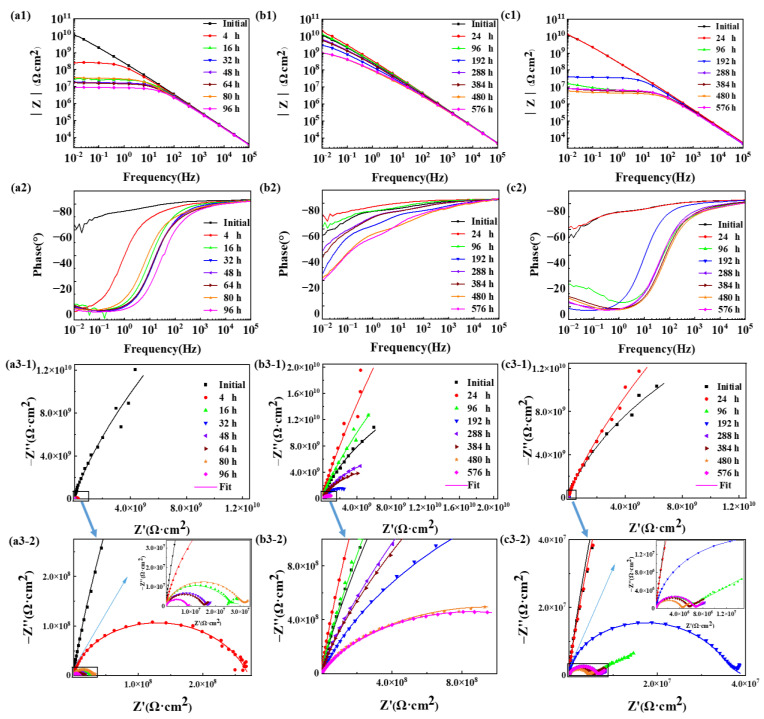
Typical Bode plots, phase angle plots and Nyquist plots for the epoxy coating samples in the E_Polarization+Immersion_ (**a1**,**a2**,**a3-1**,**a3-2**), E_Salt+Immersion+UV_ (**b1**,**b2**,**b3-1**,**b3-2**) and E_Salt+Polarization+Immersion+UV_ (**c1**,**c2**,**c3-1**,**c3-2**) tests at initial time (■) and after 4 h/24 h (●), 16 h/96 h (▲), 32 h/192 h (▼), 48 h/288 h (◀), 64 h/384 h (▶), 80 h/480 h (★) and 96 h/576 h (◆).

In Figure 2, the impedance spectra presented as Bode plots, phase angle plots and Nyquist plots of the epoxy coating samples during the 96 h/576 h (24 cycles) in the three accelerated tests were presented. At the initial time, the impedance values of the samples in Bode plots at 0.01 Hz were more than 1.2 × 10^10^ Ω·cm^2^ in Figure 2(a1,b1,c1), significantly above the threshold impedance of 1.0 × 10^7^ Ω·cm^2^. The phase angle approached near −90° over the majority of the measuring frequency range in Figure 2(a2,b2,c2). The Nyquist plot displayed in Figure 2(a3-1,a3-2,b3-1,b3-2,c3-1,c3-2) exhibited only a single time constant (one large semicircle). Hence, the epoxy coating samples exhibited excellent corrosion resistance at first. 

The impedance spectrum of the three groups of samples exhibited a distinct variation trend that can be categorized into three stages. In the 16 h/96 h (first four cycles), in the E_Polarization+Immersion_, the entire impedance (see Figure 2(a1)) decreased sharply after 4 h (one cycle), which changed from 1.28 × 10^10^ Ω·cm^2^ to 2.62 × 10^8^ Ω·cm^2^, while the region of the frequency in the phase angle (see Figure 2(a2)) near −90° drastically narrowed. And then, the semicircle in the Nyquist plot (see Figure 2(a3-1,a3-2)) shrunk rapidly, and two semicircles (two time constant) appeared after 16 h (four cycles), whereas in the E_Salt+Immersion+UV_, the impedance modulus at 0.01 Hz (see Figure 2(b1)) even reached from 1.24 × 10^10^ Ω·cm^2^ to 2.00 × 10^10^ Ω·cm^2^ after 24 h (one cycle) instead. The phase angle (see Figure 2(b2)) from the high frequency to the low frequency was nearer –90° in all the 96 h (four cycles). The radius of the semicircle in the Nyquist plot (see Figure 2(b3-1,b3-2)) also added vaguely. In the E_Salt+Polarization+Immersion+UV_, the lowest frequency impedance (see Figure 2(c1)) increased a little up from 1.21 × 10^10^ Ω·cm^2^ to 1.27 × 10^10^ Ω·cm^2^ after 24 h (one cycle) and then decreased keenly after 96 h (four cycles). The phase angle (see Figure 2(c2)) had no significant change after 24 h (one cycle), but then lost the majority of the measuring frequency range near −90° after 96 h (four cycles). The semicircle in the Nyquist plot (see Figure 2(c3-1,c3-2)) was also extended slightly after one cycle. The Warburg impedance even appeared in the Nyquist plot after 96 h (4 cycles).

From 16 h/96h (4 cycles) to 48 h/288 h (12 cycles), in the E_Polarization+Immersion_, the total impedance (see Figure 2(a1)) shrunk further and reduced to 1.81 × 10^7^ Ω·cm^2^ after 48 h (12 cycles). The range of the frequency in the phase angle (see Figure 2(a2)) near −90° became narrower rarely, and the radius of the two semicircles (see Figure 2(a3-1,a3-2)) tapered. In the E_Salt+Immersion+UV_, the impedance modulus (see Figure 2(b1)) at 0.01 Hz dropped only one magnitude after 192 h (8 cycles) and then picked up a bit after 288 h (12 cycles). The area of the frequency in the phase angle (see Figure 2(b2)) near − 90 ° lessened after 192 h (8 cycles), and then accrued a little after 288 h (12 cycles). Although the radius of the semicircle (see Figure 2(b3-1,b3-2)) decreased, it was still at a large value. In the E_Salt+Polarization+Immersion+UV_, the lowest frequency impedance (see Figure 2(c1)) raised after 192 h (8 cycles) compared to that after 96 h (4 cycles), but then lowered to 8.39 × 10^6^ Ω·cm^2^ below to 1.0 × 10^7^ Ω·cm^2^ after 288 h (12 cycles), which meant the sample began to lose its protective property. The scope of the frequency (see Figure 2(c2)) in the phase angle near −90° widened to a certain extent after 192 h (8 cycles) in comparison with that after 96 h (4 cycles), and then went back to the level as that after 96 h (4 cycles). The semicircle (see Figure 2(c3-1,c3-2)) also had the same trend, which expanded severalfold after 192 h (8 cycles) and returned to a similar size as that after 96 h (4 cycles).

In the last testing time, in the E_Polarization+Immersion_, the total impedance (see Figure 2(a1)) became closer to 1.0 × 10^7^ Ω·cm^2^ and was less than it completely after 96 h (24 cycles). The phase angle (see Figure 2(a2)) near −90° kept within a narrower range, and the value fell to −4° at 0.01 Hz. The tendency of the semicircle (see Figure 2(a3-1,a3-2)) was the same as the total impedance, which had no emerging Warburg impedance. In the E_Salt+Immersion+UV_, the impedance modulus (see Figure 2(b1)) at 0.01 Hz declined a small amount and still maintained in the 10^9^ Ω·cm^2^ lever after 576 h (24 cycles). The district of the phase angle (see Figure 2(b2)) near −90° did not cut down too much. As the same changing situation, the radius of the semicircle (see Figure 2(b3-1,b3-2)) remained at a large value, whose number was also one (one time constant). In the E_Salt+Polarization+Immersion+UV_, the lowest frequency impedance (see Figure 2(c1)) mainly concentrated in 10^6^ Ω·cm^2^ and not far away from 1.0 × 10^7^ Ω·cm^2^. The range of the phase angle (see Figure 2(c2)) near −90° was little different compared with that after 288 h (12 cycles). The semicircle (see Figure 2(c3-1,c3-2)) continued to become smaller without the Warburg impedance emerging again. 

Based on the cycles of the entire impedance being less than 1.0 × 10^7^ Ω·cm^2^, the number of the cycles of the epoxy coating samples within the three accelerated methods was 24 cycles (in the E_Polarization+Immersion_, 96 h), above 24 cycles (in the E_Salt+Immersion+UV_, above 576 h) and 12 cycles (in the E_Salt+Polarization+Immersion+UV_, 288 h). Obviously, just as far as the corrosion rates were solely attributed to the coating systems, these three tests could be ranked in a specific order: E_Polarization+Immersion_, E_Salt+Polarization+Immersion+UV_ and E_Salt+Immersion+UV_. However, for the accelerated efficiency from the numbers of testing cycles, the ranks were in the following order: E_Salt+Polarization+Immersion+UV_, E_Polarization+Immersion_ and E_Salt+Immersion+UV_.

### 3.2. Surface Morphology

**Figure 3:**
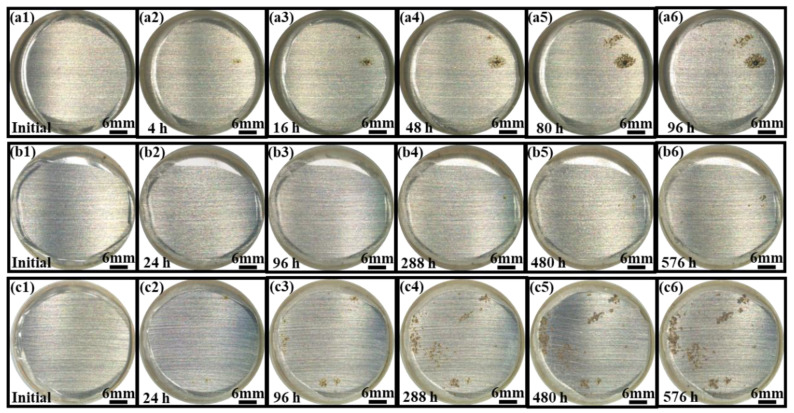
The surface morphologic images for the epoxy coating samples changing within 96 h/576 h (24 cycles) under the E_Polarization+Immersion_ (**a1**–**a6**), E_Salt+Immersion+UV_ (**b1**–**b6**) and E_Salt+Polarization+Immersion+UV_ (**c1**–**c6**) tests.

The surface morphologies of the epoxy coating samples with different cycles in the three accelerated tests are depicted in Figure 3(a1–c6). At the initial time, the surfaces of the coatings were transparent and intact with no corrosion pits (see Figure 3(a1,b1,c1)). However, it was quickly observed that there were one or two small corrosion spots that appeared on the carbon steel substrate surface in the E_Polarization+Immersion_ after 4 h (one cycle) and the E_Salt+Polarization+Immersion+UV_ after 24 h (one cycle). As time went on, the corrosion spots gradually expanded to be a clearly corroded area. At the same time, some new corrosion spots arose on the other carbon steel substrate region. The corroded area in the E_Salt+Polarization+Immersion+UV_ after 288 h (12 cycles) was obviously larger than that in the E_Polarization+Immersion_ after 48 h (12 cycles) (see Figure 3(a4,c4)). It meant that the corrosion in the E_Salt+Polarization+Immersion+UV_ was more serious than that in the E_Polarization+Immersion_. The corroded area was further enlarged and the extent of corrosion was more serious in the E_Polarization+Immersion_ after 96 h (24 cycles) and in the E_Salt+Polarization+Immersion+UV_ after 576 h (24 cycles). In particular, only a small corrosion pit came out in the E_Salt+Immersion+UV_ until 576 h (24 cycles).

### 3.3. Color Changes 

**Figure 4:**
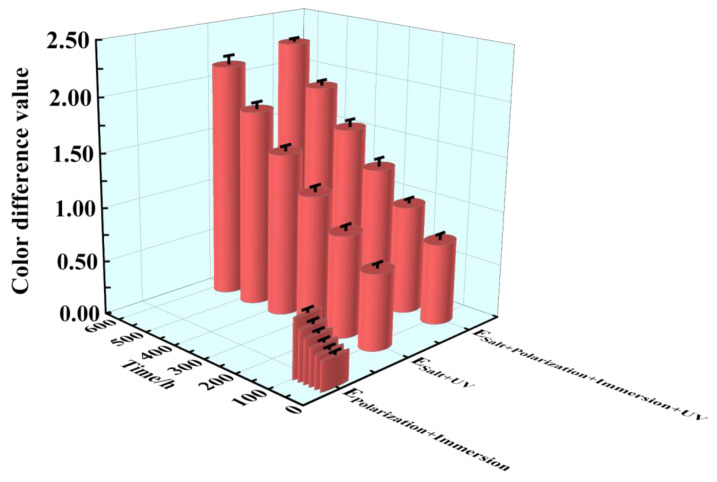
The color difference for the epoxy coating samples changing within 96 h/576 h (24 cycles) in the E_Polarization+Immersion_, E_Salt+Immersion+UV_ and E_Salt+Polarization+Immersion+UV_ tests.

The destruction condition of the epoxy coating film could be measured with a color change, which was obtained by calculating the color difference ΔE of the sample between different cycles and the beginning. In Figure 4, the values of the color difference ΔE under the three accelerated tests were exhibited within 96 h/576 h (24 cycles). All the color difference ΔE values increased with the progress of the cycles. The biggest ΔE value was 2.33 below 5.0, which signified that the whole epoxy coating film of the samples were less broken within 96 h/576 h (24 cycles). However, according to the trend of the color difference ΔE, the final average values were 0.54, 2.16 and 2.27. It was revealed that the damage condition of the epoxy coating samples in the E_Salt+Polarization+Immersion+UV_ and the E_Salt+Immersion+UV_ was more severe than that in the E_Polarization+Immersion_, and that in the E_Salt+Polarization+Immersion+UV_ was slightly more acute than that in the E_Salt+Immersion+UV_ for the duration.

### 3.4. Surface Roughness

**Figure 5:**
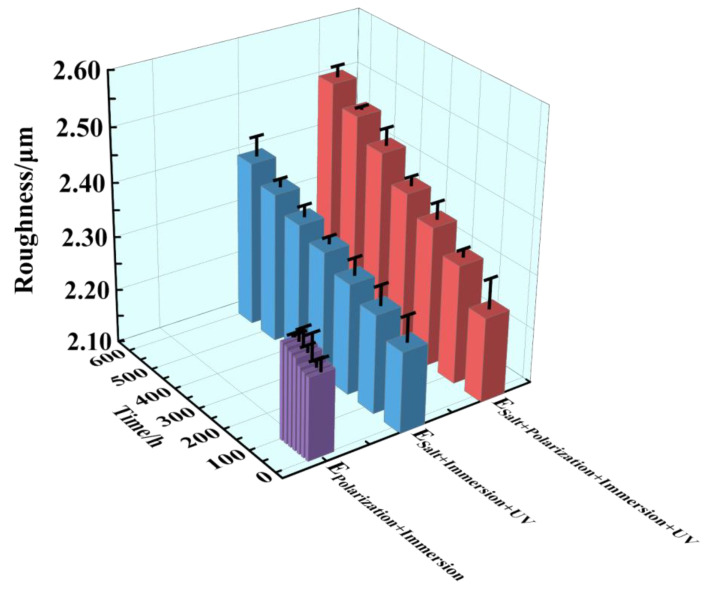
The roughness for the epoxy coating samples changing within 96 h/576 h (24 cycles) in the E_Polarization+Immersion_, E_Salt+Immersion+UV_ and E_Salt+Polarization+Immersion+UV_ tests.

In Figure 5, the surface roughness of the three accelerated tests is depicted, illustrating the extent of destruction observed on the epoxy coating surface layer. The surface roughness average values of all the epoxy coating samples were 2.25 at the initial time, which increased gradually with the extension of time. After 96 h/576 h (24 cycles), the surface roughness average values of the samples in the three tests reached 2.27, 2.41 and 2.51. Among them, the surface roughness in the E_Polarization+Immersion_ was similar, which indicated that almost no damage occurred in the epoxy coating samples. In addition, the changing range of the surface roughness in the E_Salt+Polarization+Immersion+UV_ and the E_Salt+Immersion+UV_ was larger, which meant that a certain amount of damage had occurred in the two tests. And the broken condition in the E_Salt+Polarization+Immersion+UV_ was a bit nastier than that in the E_Salt+Immersion+UV_, constantly.

### 3.5. Chemical Structure

**Figure 6:**
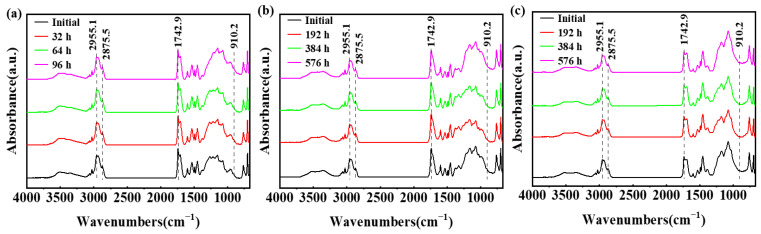
The FT-IR spectra for the epoxy coating samples changing within 96 h/576 h (24 cycles) in the E_Polarization+Immersion_ (**a**), E_Salt+Immersion+UV_ (**b**) and E_Salt+Polarization+Immersion+U_ tests (**c**).

The FT-IR micro-spectroscopy technique can provide an in situ test function and improve the detection sensitivity. Hence, investigating the structural modifications of epoxy coatings under ultraviolet irradiation is a commonly employed approach to examine the degradation of such coatings. As shown in Figure 6, the peak positions of the functional groups from the epoxy coating sample were analyzed during three accelerated tests. The characteristic peaks corresponding to C-H stretching vibration and -CH_2_ symmetric stretching vibration were observed at 2955.1 cm^−1^ and 2875.5 cm^−1^, respectively. The stretching vibration peak associated with the C=O bond appeared at 1742.9 cm^−1^, while the characteristic peak of the epoxy group was identified at 910.2 cm^−1^. Throughout the entire process, both the intensity and position of these major peaks of the functional groups remained relatively stable, indicating minimal alteration in their absorption characteristics.

To further comprehend the microscopic changes in chemical structure of the epoxy coating during the three tests, the epoxy group area ratio and C=O/C-H band area ratio were studied. Because the post-curing of the epoxy groups and dissociation of alkyl groups may still occur during the aging process of the epoxy coatings, this involves the following major chemical reactions: Equations (1) and (2).

(1) $$\begin{array}{l} R_{1}–{\mathrm{CH}}_{2}–{\mathrm{NHR}}_{2}+{\mathrm{CH}}_{2}{\left(O\right)}_{{\mathrm{CHR}}_{3}}\\ \to \\ R_{1}–{\mathrm{CH}}_{2}–{\mathrm{NR}}_{2}–{\mathrm{CH}}_{2}{\mathrm{CHR}}_{3}–OH \end{array}$$

(2) $$\begin{array}{l} R_{4}–C(=O)–NH–{\mathrm{CH}}_{2}–{\mathrm{CH}}_{2}–R_{5}\\ \frac{hv}{several}\overset{O_{2}}{\underset{steps}{\to }}\\ R_{4}–C(=O)–NH–CH(O•)–{\mathrm{CH}}_{2}–R_{5}\\ \to \\ R_{4}–C(=O)–NH–CH(=O)+•{\mathrm{CH}}_{2}–R_{5} \end{array}$$

**Figure 7:**
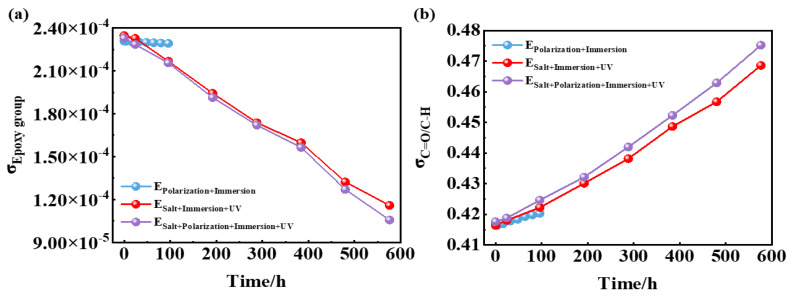
Time dependence of the proportion for epoxy group band area to the total peak area (**a**), and the band area ratio of C=O to C-H groups (**b**) within 96 h/576 h (24 cycles) of the E_Polarization+Immersion_, E_Salt+Immersion+UV_ and E_Salt+Polarization+Immersion+UV_ tests.

In the Equation (1) reaction, the epoxy groups were consumed, whose band area proportion could be used to revealed the post-curing degree of the epoxy coatings. In the Equation (2) reaction, the C=O groups were newly formed, while the C-H was reduced. Therefore, the destruction level of the epoxy coatings during the aging process could be quantified by the ratio of band areas between C=O and C-H. In Figure 7a, there has been a gradual decrease in the proportion of epoxy group band area to the total peak area. Notably, E_Salt+Polarization+Immersion+UV_ exhibited the most significant reduction, slightly surpassing E_Salt+Immersion+UV_ and significantly exceeding E_Polarization+Immersion_. These findings indicate that there is a post-curing phenomenon occurring in epoxy coatings under ultraviolet irradiation. In Figure 7b, the ratio of C=O to C-H increases with an increasing number of cycles, further confirming decomposition within the polymer coated with epoxy. The rise in C=O to C-H ratio is much more pronounced in E_Salt+Polarization+Immersion+UV_ and E_Salt+Immersion+UV_ compared to E_Polarization+Immersion_, suggesting that UV radiation could expedite the deterioration process of epoxy-coated polymers.

### 3.6. Adhesion Analysis

**Figure 8:**
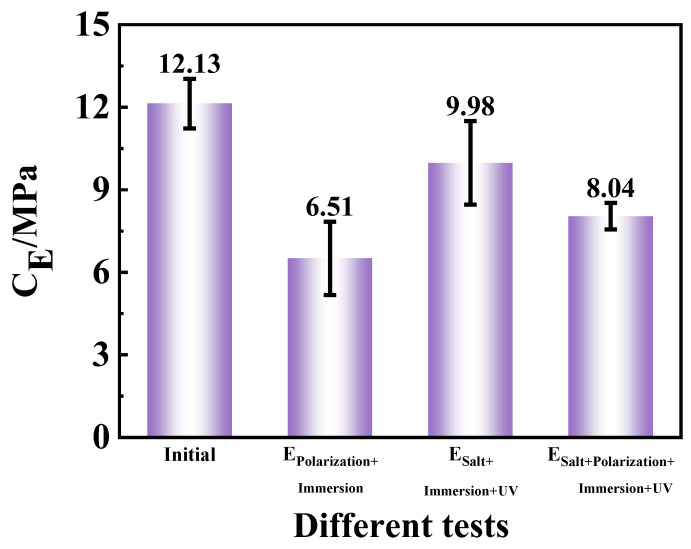
The adhesion for the epoxy coating samples at the initial time and after 96 h/576 h (24 cycles) of the E_Polarization+Immersion_, the E_Salt+Immersion+UV_ and the E_Salt+Polarization+Immersion+UV_ tests.

Adhesion of the coatings is a significant index to evaluate the binding performance between the coatings and the metallic substrates. It could indirectly reflect the extent of corrosion damage on coated samples, as reduced adhesion was typically caused by blistering at the interface between the coating and carbon steel or deadhesion due to corrosion. In Figure 8, the adhesion in the three accelerated tests decreased in different amplitude compared with the initial samples. The adhesion values of the E_Polarization+Immersion_, the E_Salt+Immersion+UV_ and the E_Salt+Polarization+Immersion+UV_ dropped down from 12.13 MPa to 6.51 MPa, 9.98 MPa and 8.04 MPa, respectively. These results indicated that there was serious corrosion occurring at the interface between the coating and substrate in E_Polarization+Immersion_ and E_Salt+Polarization+Immersion+UV_ tests, while less corrosion damage was observed in E_Salt+Immersion+UV_.

### 3.7. SEM Morphologies and EDS Analysis

**Figure 9:**
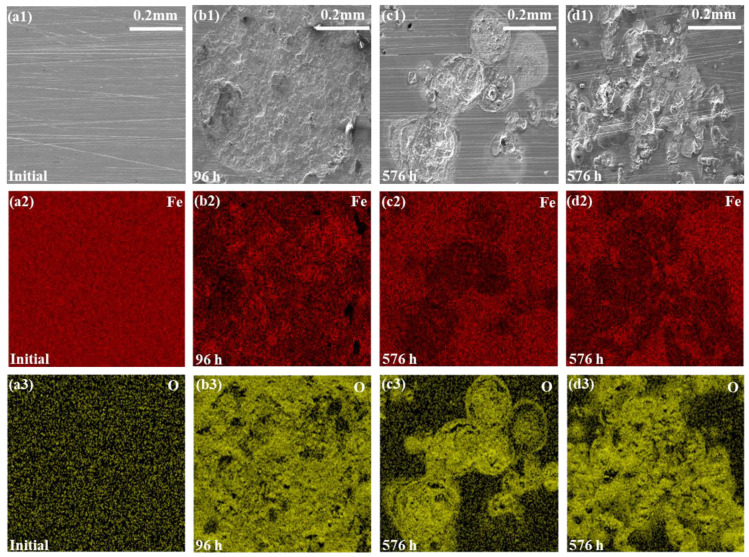
SEM images and EDS results for the epoxy coating samples at the initial (**a1**–**a3**) and after 96 h/576 h (24 cycles) in the E_Polarization+Immersion_ (**b1**–**b3**), the E_Salt+Immersion+UV_ (**c1**–**c3**) and the E_Salt+Polarization+Immersion+UV_ (**d1**–**d3**).

The carbon steel substrate morphologies and the composition of the corrosion products were analyzed by SEM with EDS at the destruction sites in the back of the adhesion measurement. The SEM morphologies were from the initial surface and the most severe corrosion region of the carbon steel substrate. In Figure 9, the corrosion products in the corroded area predominantly consisted of iron and oxygen elements. This observation suggested the formation of iron-containing oxides within the affected region [54,55]. Among them, the O element was mainly distributed in the corrosion damage areas, which was obviously different from that in the non-corrosive region. Although the Fe element consumed all the corrosion damage areas, the distribution of the Fe element in the corrosion damage area was also different from that in the non-corrosive region. 

**Figure 10:**
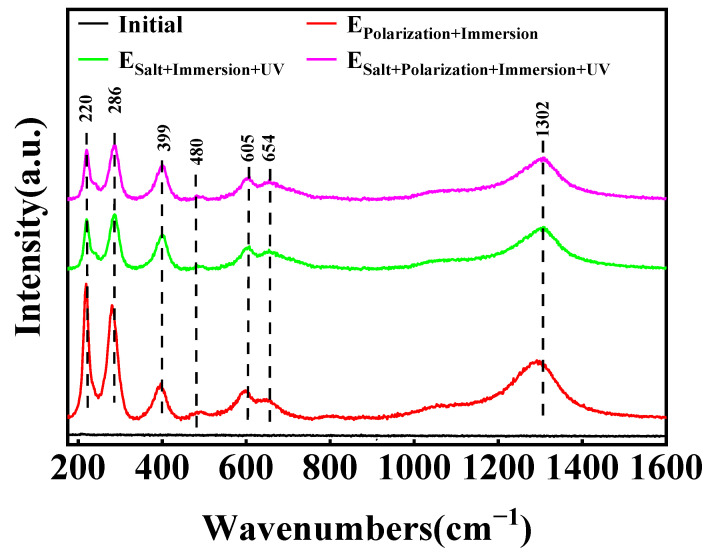
Raman spectra of the corrosion products for the epoxy coating/carbon steel samples at the initial time and 96 h/576 h (24 cycles) in the E_Polarization+Immersion_, E_Salt+Immersion+UV_ and E_Salt+Polarization+Immersion+UV_ tests.

The Raman spectra of the corrosion products (see Figure 10) reveal that in the destructed site after the adhesion test, the peak values at 220, 286, 399, 480, 605 and 1302 cm^−1^ can be put down to α-Fe_2_O_3_, while the peak value at 654 cm^−1^ should be imputed to Fe_3_O_4_ [56,57]. These characteristic peaks further indicate the formation of iron oxides during the metamorphic process of the samples.

**Figure 11:**
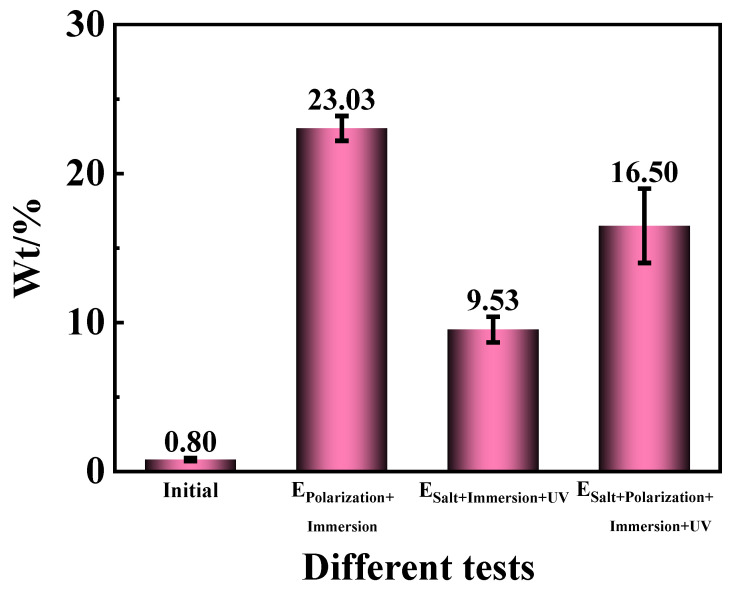
The detected mass contents of Fe in the epoxy coating samples at the initial and 96 h/576 h (24 cycles) in the E_Polarization+Immersion_, E_Salt+Immersion+UV_ and E_Salt+Polarization+Immersion+UV_ tests.

The higher the iron oxide content in the corrosion zone, the greater the presence of the oxygen (O) element, indicating a more severe degree of corrosion due to increased oxidation of O into iron oxide. In order to reveal the specific O element content of the entire area in the morphologies, the EDS test [58] was implemented. The results in Figure 11 exhibited that the original O content on the carbon steel surface was about 0.80 wt.%. In the three accelerated tests of the E_Polarization+Immersion_, the E_Salt+Immersion+UV_ and the E_Salt+Polarization+Immersion+UV_ after 96 h/576 h (24 cycles), the O content rose to 23.03 wt.%, 9.53 wt.% and 16.50 wt.%, respectively. It meant that the corrosion degree of the carbon steel substrate in the E_Polarization+Immersion_ was most severe. The deterioration in the E_Polarization+Immersion_ and the E_Salt+Polarization+Immersion+UV_ was more serious than that in the E_Salt+Immersion+UV_.

## 4. Discussion

### 4.1. Corrosion-Acceleration Mechanism of E_Polarization+Immersion_

**Figure 12:**
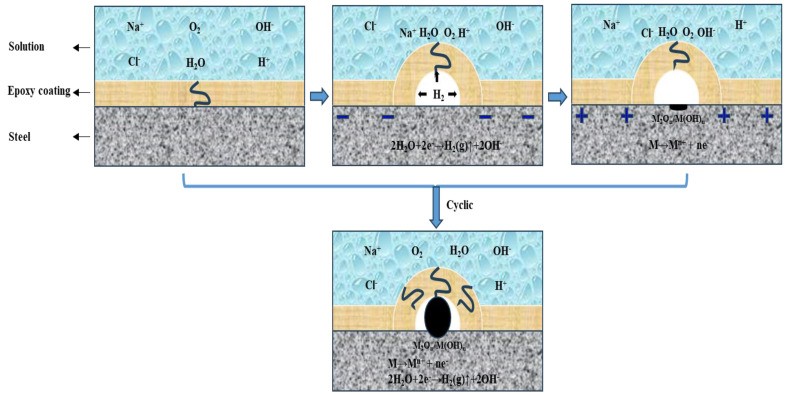
Schematic illustration of corrosion failure mechanism of the epoxy coating samples undergoing the E_Polarization+Immersion_.

The epoxy coating samples immersed in 3.5 wt.% NaCl solution had a constant cathodic reaction:

2H_2_O + 2e^−^ → 2OH^−^ + H_2_↑ and/or O_2_ + 2H_2_O + 4e^−^ → 4OH^−^ (3)

and anodic reaction:

M → M^n+^ + ne^−^ (4)

involved in the corrosion failure process. In the E_Polarization+Immersion_, the corrosion damage of the samples was accelerated by the alternating cathodic and anodic polarization with symmetrical positive and negative electric charges. The detailed corrosion acceleration mechanism [48] can be summarized as follows and schematically depicted in Figure 12:

(a) Firstly, the O_2_ and cations such as H^+^ and Na^+^ in 3.5 wt.% NaCl solution are transported in the cathodic DC step to the metallic substrate through the micro defects and pores in the epoxy coating. Meanwhile, the cathodic reaction (reaction (3)) occurring on the metallic substrate surface is accelerated by the cathodic polarization, resulting in fast formation of H_2_ and OH^−^. The evolved H_2_ pushes the coating film away from the substrate, forming a gaseous bubble between the epoxy coating film and the carbon steel substrate. The generated OH^−^ can also impair the bonding strength between the coating film and the substrate.
(b) Secondly, immediately after the cathodic polarization is the anodic DC step. The anions Cl^−^ and OH^−^ are transported in the 3.5 wt.% NaCl solution to the metallic substrate through the micro defects and pores in the epoxy coating film. At the same time, the substrate metal is anodically dissolved at a high rate on account of the accelerated anodic reaction (reaction (4)). Some of the dissolved metal cations combine with OH^−^ to form hydroxides/oxides.
(c) Thirdly, in the relaxation stage, the corrosion products of
  2M^n+^ + 2nOH^−^ → 2M(OH)_n_ → M_2_O_n_ + nH_2_O (5)
  mainly the hydroxides/oxides, are further produced duo to the corrosion of the substrate metal in the presence of the H_2_O and Cl^−^.

According to this model, under the alternating cathodic and anodic polarization, H^+^, Na^+^, Cl^−^ and H_2_O and O_2_ would more quickly arrive at the surface of the epoxy coating/carbon steel substrate than under a static immersion, which could lower the impedance (see Figure 2(a1,a3-1,a3-2)). The formed hydroxides/oxides could spread rapidly along the metallic substrate, which could be seen in the surface morphologic images (see Figure 3). The generated products containing H_2_ bubbles, OH^−^ and hydroxides/oxides dramatically whittled the adhesion between the epoxy coating film and the carbon steel substrate, pushing and wedging the coating film apart off the substrate. Therefore, the coating delamination (see Figure 7) and the carbon steel substrate corrosion (see Figure 9 and Figure 11) in the E_Polarization+Immersion_ were the most serious.

Because the cathodic polarization and anodic polarization were alternately applied with symmetrical positive and negative electric charges, the cathodic reaction (reaction (3)) and anodic reaction (reaction (4)) were equally accelerated in each cycle. It is the most important feature of a natural corrosion process that the anodic process is always equally coupled by the cathodic process. Therefore, the E_Polarization+Immersion_ test kept the critical feature of a corrosion process, while the anodic and cathodic processes were equally accelerated (i.e., the corrosion process is accelerated). In other words, the accelerated test did not alter the corrosion mechanism of the epoxy coating samples in the immersion environment. 

Similar research was carried out in our last study [48], which was executed once in a 24 h period. Under the premise that the OCP of the sample returned to the stable state after each polarization, the test number was added in the E_Polarization+Immersion_ test, resulting in the failure rate being accelerated to a certain extent. 

### 4.2. Corrosion-Acceleration Mechanism of E_Salt+Immersion+UV_

The E_Salt+Immersion+UV_ test included salt spraying, drying, humidifying, salt-immersion and UV irradiation steps. It simulated the ocean splash environment. An epoxy coating usually degrades with time after it is exposed to the simulated environment of a multi-factor coupling-accelerated test. Salt spray can accelerate the penetration rate of water and corrosive media in the organic coating, thereby shortening the time to reach the metal substrate [59]. The alternation of drying and humidifying combined with the alternation of temperature and salinity in the test could also inflict a swelling–shrinking effect on the organic coating, and consequently weaken or even break the coating [60,61]. The photo-oxidation resulting from UV light may trigger and initiate a chain scission of polymer molecules [62,63]. Among that, parts of the same conclusions about disruptive behavior have been found in our group’s previous research [17]. However, in the E_Salt+Immersion+UV_ test, an interesting phenomenon was observed, that the impedance of the epoxy coating did not decrease in 96 h (in the first four cycles), but was improved (see Figure 2(b1,b3-1,b3-2)). This might be caused by the so-called post-curing process, in which the UVA irradiation further cured the epoxy (see reaction (1) and Figure 7a), and the epoxy coating molecules became denser with more UVA-inducing cross-links. This post-curing effect might to some degree balance the UVA-irradiation-induced degradation of the epoxy coating in the top layer (see reaction (2) and Figure 7b). Hence, the overall corrosion resistance of the samples film was modified in the early stage, resulting in smaller corrosion areas on the metallic substrate (see Figure 3(b1–b3)). Obviously, the post-curing effect gradually ceased and the UVA-irradiation-induced damage accumulated with time, the protection performance of the coating eventually deteriorated. It was also the reason that the changes in color and surface roughness became more significant with time later on (see Figure 4 and Figure 5).

**Figure 13:**
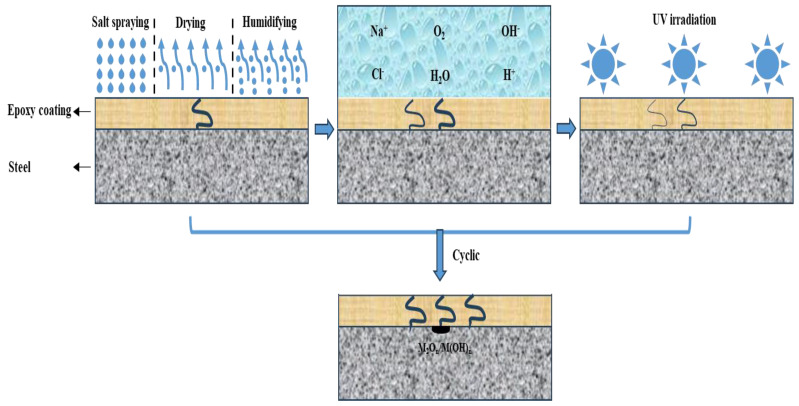
Schematic illustration of corrosion failure mechanism of the epoxy coating samples undergoing the E_Salt+Immersion+UV_.

As in the E_Salt+Immersion+UV_, no alternating cathodic and anodic polarization steps were involved to accelerate the destruction of the epoxy coating, the overall damage rate of the epoxy coating was slower. This could also result in less loss of ultimate adhesion of the coating and less severe corrosion of the metallic substrate (see Figure 8, Figure 9 and Figure 11). However, owing to the coupling failure effects from the salt spraying, drying, humidifying, high temperature, salt-immersion and UV irradiation steps, the decline rate of the impedance mode and the corrosion area of the metal substrate were higher than those in the test that only combined with salt-immersion and UV irradiation steps at the same testing period, which could be seen in our group’s last research [48]. According to the above process, the detailed corrosion acceleration mechanism can be summarized below and schematically illustrated in Figure 13.

### 4.3. Corrosion-Acceleration Mechanism of E_Salt+Polarization+Immersion+UV_

The E_Salt+Polarization+Immersion+UV_ is composed of the E_Polarization+Immersion_ with the E_Salt+Immersion+UV,_ in which the corrosion of the carbon steel substrate is mainly accelerated by the cathodic and anodic polarization. The corrosion destruction of the epoxy coating samples in the test differed from that in the E_Salt+Immersion+UV_. The anti-corrosion performance of the epoxy coating was enhanced a little bit after 24 h (in the first cycle). The reason was also that the enhancement effect from the post-curing process in the epoxy coating was stronger than the damage effect from the coupling process of the salt spraying, drying, humidifying, high temperature, salt immersion combined with the cathodic and anodic polarization and the UVA irradiation. And then descended rapidly in the later cycles as indicated by the decreasing impedance (see Figure 2(c1,c3-1,c3-2)). This indicated that the damage effect began to be greater than the enhancement effect, and the damage effect promoted the penetration and transmission of water and corrosive media in the coating. Especially, the appearance of the Warburg impedance in the Nyquist plot after 96 h (4 cycles) implied that the corrosive medium had reached the metallic substrate surface and caused obvious electrochemical corrosion there. The detail corrosion damage could be observed in Figure 3(c3). The accidentally enhanced impedance at 192 h (the 8th cycle) (see Figure 2(c1,c3-1,c3-2)) might be a result of the blockage of the micro pores and defects in the epoxy coating because of the accumulated corrosion products from the carbon steel substrate. After 288 h (12 cycles), the epoxy coating lost its protective property, and the impedance decreased below 1.0 × 10^7^ Ω·cm^2^. Because the damage effect from the coupling process of the salt spraying, drying, humidifying, high temperature, salt immersion combined with the cathodic and anodic polarization surpassed that in the process of only cathodic and anodic polarization, and was stronger than the coupling process of the only salt immersion combined with the cathodic and anodic polarization [48], the E_Salt+Polarization+Immersion+UV_ had a higher corrosion acceleration effect than the other two tests in the same number of cycles (but different periods), as a whole. 

**Figure 14:**
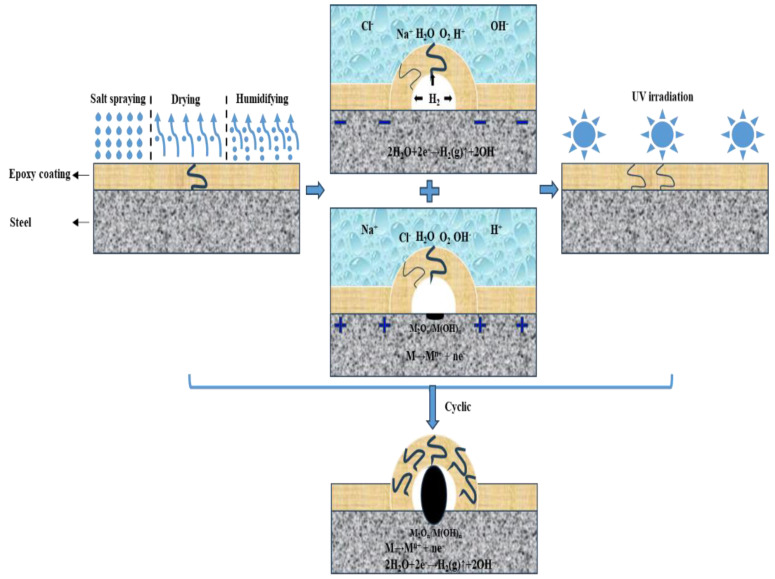
Schematic illustration of corrosion failure mechanism of the epoxy coating samples undergoing the E_Salt+Polarization+Immersion+UV_.

Another interesting phenomenon was that the changes in color, surface roughness and the ratio of C=O to C-H in the coating during the E_Salt+Polarization+Immersion+UV_ were greater than those in the E_Salt+Immersion+UV_. There might be two reasons for the greater changes. The first one is that the accumulation of corrosion products at the surface between the coating and the carbon steel substrate during the E_Salt+Polarization+Immersion+UV_ caused serious deformation of the coating, resulting in more micro-defects in the coating and an expanded surface area exposed to ultraviolet light. The second reason is that the deposited corrosion products on the metallic substrate might absorb more heat locally from the ultraviolet light and then release to heat the epoxy coating in the local area, which could also accelerate the degradation of the coating. According to the above process, the detailed corrosion acceleration mechanism can be summarized below and schematically illustrated in Figure 14.

### 4.4. Corrosion Acceleration Effects of the Three Tests

From what has been discussed above, the corrosion acceleration effects of the three tests were compared. In the E_Polarization+Immersion_, because the penetration and transmission rate of water and corrosive media in the coating were accelerated by the alternating cathodic and anodic polarization with symmetrical positive and negative electric charges, the time of arriving at the carbon steel substrate was shortened, resulting in the stripping of the epoxy coating at the interface between the carbon steel substrate and the coating, which was conducive to the accelerated corrosion rate of the carbon steel substrate. It could be seen that the impedance values of the epoxy coating samples descended rapidly in a short time. The corrosion area of the carbon steel substrate gradually expanded with the extension of the testing time. On account of no drying, humidifying and UVA irradiation, the chemical performances of the epoxy coating were merely lost in the test.

There were two effects competing with each other in the E_Salt+Immersion+UV_. The one was a damage effect from the coupling process of the salt spraying, drying, humidifying, high temperature, salt immersion and UVA irradiation, leading to the acceleration of the penetration and transmission rate of water and corrosive media in the coating, the weakening or even breaking of the polymer and the chain scission of polymer molecules. The other one was an enhancement effect from the post-curing process by the UVA irradiation, causing the molecular structure to become denser. Because the test time was not long, the enhancement effect was more dominant than the damage effect, contributing to most degradation of the samples concentrated in the polymer surface layer of the coating, and the damage of the carbon steel substrate was less. However, as the testing time went on, the situation reversed along with a gradual decline in the anti-corrosion property of the samples. 

To the E_Salt+Polarization+Immersion+UV_, it was combined with the E_Polarization+Immersion_ on the basis of the E_Salt+Immersion+UV_. The damage effect was greatly strengthened by the new coupling process of the salt spraying, drying, humidifying, high temperature, salt immersion combined with the cathodic and anodic polarization and the UVA-irradiation-induced degradation, which was superior to the enhancement effect from the post-curing process at the early time after 24 h (the first cycle). Moreover, with the progress of the testing time, the damage effect under the new coupling process would continue to be intensified, which was far greater than the enhancement effect, causing the failure rate of the samples to be unremittingly accelerated. Therefore, the degradation rate of the samples in E_Salt+Polarization+Immersion+UV_ was the fastest from the 4th cycle to the 24th cycle, whose corresponding time was from 96 h to 576 h.

## 5. Conclusions

(1) Under the immersion environment during the E_Polarization+Immersion_, the alternating cathodic and anodic polarization with symmetrical positive and negative electric charges can dramatically accelerate both the cathodic and anodic reactions. The destruction of the epoxy coating systems was rapidly accelerated, which lost its anti-corrosion property after 24 cycles equal to 96 h. The main damage sites were the corrosion of metal substrate, while there was a mere loss of the chemical performances of the epoxy coating samples.
(2) The E_Salt+Immersion+UV_ consists of salt spray (containing wet, dry and humid), immersion and UV irradiation, which could preferably simulate the ocean splash zone. The destruction effect from the salt spraying, drying, humidifying, high temperature, salt immersion and UVA irradiation competed with the enhancement effect from the post-curing process by the UVA irradiation within the test time. The damage effect was weaker than the enhancement effect at early time, resulting in the entire impedance of the coating samples being improved about 1.6 times more than that in the initial time. As the testing time progressed, the situation reversed along with a gradual anti-corrosion performance decline in the samples.
(3) The E_Salt+Polarization+Immersion+UV_ is a combination of E_Polarization+Immersion_ and E_Salt+Immersion+UV_. The coupling process of the salt spraying, drying, humidifying, high temperature, salt immersion combined with the cathodic and anodic polarization and the UVA-irradiation-induced degradation causes the acceleration of the penetration and transmission rate of water and corrosive media in the coating, the weakening or even break of the polymer and the chain scission of polymer molecules to occur together, whose damage effect is much greater than the enhancement effect from the post-curing process. It had the strongest damage-acceleration effect in the E_Salt+Polarization+Immersion+UV_ than the other two tests.

## Data Availability

Data are contained within the article.
